# Nitrato(5,10,15,20-tetra­phenyl­porphinato)manganese(III)–benzene–*n*-hexa­ne (2/1/1)

**DOI:** 10.1107/S2414314622003868

**Published:** 2022-04-22

**Authors:** Hongli Cao, Junwen Wang, Jianfeng Li

**Affiliations:** a Key Laboratory of Magnetic Molecules, Magnetic Information Materials, Ministry of Education, School of Chemical and Material Science, Shanxi Normal University, Taiyu Road, Taiyuan 030032, People’s Republic of China; b College of Materials Science and Opto-electronic Technology, CAS Center for Excellence in Topological Quantum Computation & Center of Materials Science and Optoelectronics Engineering, University of Chinese Academy of Sciences, Yanqi Lake, Huairou District, Beijing 101408, People’s Republic of China; Vienna University of Technology, Austria

**Keywords:** crystal structure, manganese(III) porphyrin, nitrate

## Abstract

The crystal structure of solvated [Mn(TPP)(NO_3_)] (TPP = 5,10,15,20-tetra­phenyl­porphyrinato, C_44_H_28_N_4_), [Mn(C_44_H_28_N_4_O_3_)(NO_3_)]·0.5C_6_H_14_·0.5C_6_H_6_, has been determined in the space group *Pccn*. The Mn^III^ atom has a distorted square-pyramidal environment, being coordinated by four pyrrole N atoms of the porphyrin ligand in the basal plane and an O atom of the nitrato ligand in the apical site. The Mn^III^ atom is displaced out of the porphyrin plane by 0.22 (4) Å with the average Mn—Np distance being 2.011 (6) Å (where Np is a porphyrin N atom). The Mn—O bond length is 2.1246 (18) Å. Two kinds of inter­molecular C—H⋯O hydrogen bonds exist in the crystal structure, with the apical nitrato ligands inter­acting with solvent mol­ecules and adjacent mol­ecules, respectively.

## Abstract 2

The central manganese(III) atom has a distorted square-pyramidal coordination environment, defined by the porphyrin N atoms in the basal plane and a nitrate O atom in the apical site.

## Graphical abstract

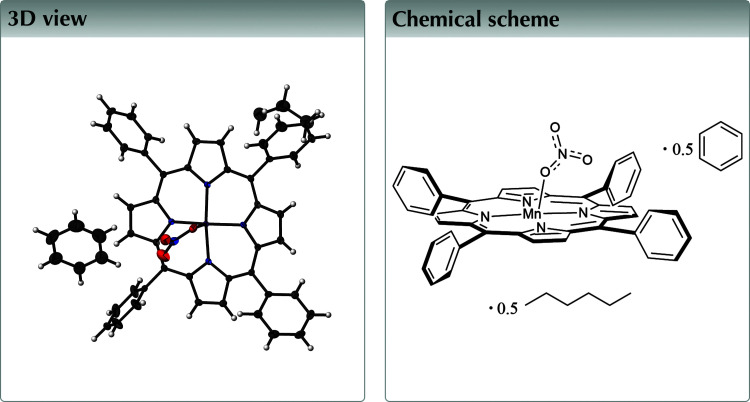

## Structure description

Inter­actions between metalloporphyrins and nitrate ligands occur in many areas of bioinorganic chemistry. Nitrates play a key role in fixing atmospheric nitro­gen into a more bioavailable form, as detailed in the nitro­gen cycle (Averill, 1996 ▸). A series of nitrate-coordinating Fe^III^ derivatives have been reported, whereby the denticity of the nitrate ligands shows differences for complexes of the general type [Fe(Por)(NO_3_)] (where Por is porphyrin). Among them, [Fe(OEP)(NO_3_)] (OEP = 2,3,7,8,12,13,17,18-octa­ethyl­porphyrinato, space group *P* 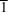 ; Ellison *et al.*, 1996 ▸), [Fe(OEP)(NO_3_)] (space group *P*2_1_/*c*, Wyllie *et al.*, 2007 ▸) and [Fe(4-Me-TTP)(NO_3_)] (TTP = 5,10,15,20-tetra­kis­(4-methyl­phen­yl)porphyrinato; Bhuyan & Sarkar, 2013 ▸) have a nitrate group monodentately binding to the central metal cation, while [Fe(TPP)(NO_3_)] (Wyllie *et al.*, 2007 ▸), [Fe(4-OMe-TPP)(NO_3_) (4-OMe-TPP = 5,10,15,20-tetra­kis­(4-meth­oxy­phen­yl)-porphyrinato; Bhuyan & Sarkar, 2013 ▸) and [Fe(TpivPP)(NO_3_)] (TpivPP = α,α,α,α-tetra­kis­(*o*-pival­amido­phen­yl)porphyrinato; Munro & Scheidt, 1998 ▸) have a nitrate group bidentately binding to the central cation. Herein, we report the structural properties of a related manganese(III) compound, *viz*. [Mn(TPP)(NO_3_)], crystallizing as a hemisolvate of benzene and *n*-hexane. In accordance with the benzene disolvate of [Mn(TPP)(NO_3_)] (Suslick & Watson, 1991 ▸), the nitrato ligand binds monodentately. The key crystal structural parameters of all the above-mentioned metalloporphyrin nitrate complexes are given in Table 1 ▸. It is seen that the average Mn—Np bond length and the Mn—O1 bond length of the title complex are 2.011 (6) and 2.1246 (18) Å, respectively, both of which are slightly longer than the values of 2.007 (9) and 2.101 (3) Å found in the structure of the triclinic benzene disolvate [Mn(TPP)(NO_3_)]·2C_6_H_6_ (Suslick & Watson, 1991 ▸).

**Figure 1:**
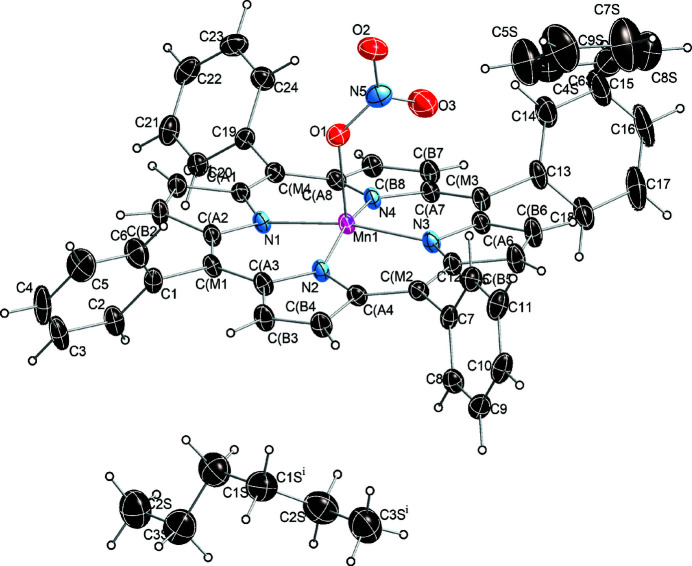
The mol­ecular structure of the title compound, drawn with displacement ellipsoids at the 50% probability level. Only one of the two orientations of the disordered benzene solvate mol­ecules is shown. [Symmetry code: (i) −*x* + 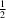 , −*y* + 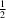 , *z*.]

**Figure 2:**
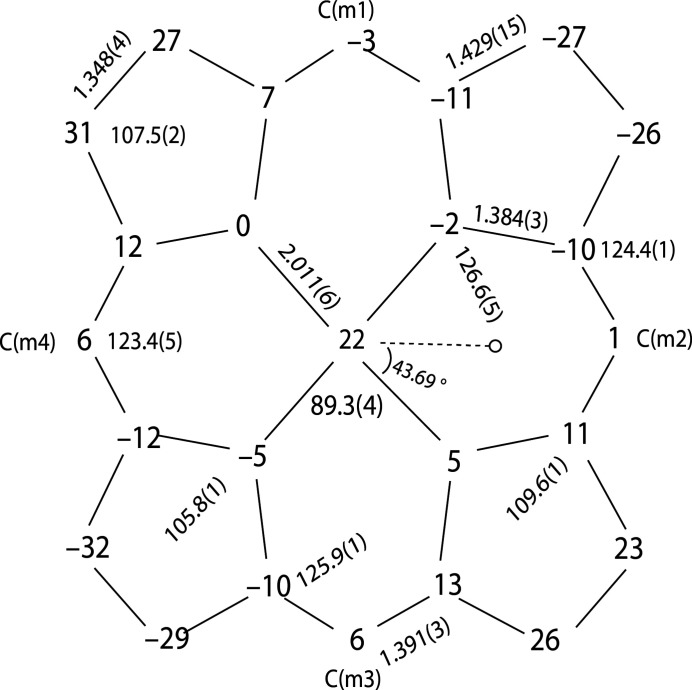
A formal diagram of the porphyrin core of the title compound. Averaged values of the chemically unique bond lengths (Å) and angles (°) are shown. The perpendicular displacements (in units of 0.01 Å) of the porphyrin core atoms from the 24-atom mean plane are also displayed. The positive numbers indicate a displacement towards the nitrate ligand, the dashed line indicates the plane of the nitrate ligand on the unhindered porphyrin side.

In the crystal structure of the title five-coordinate manganese(III) nitrate complex (Fig. 1 ▸), the asymmetric unit contains one porphyrin mol­ecule, half of a benzene solvate mol­ecule, and half of an *n*-hexane solvate mol­ecule. The Mn1^III^ atom has a distorted square-pyramidal environment, defined by the four pyrrole N atoms of the porphyrin ligand in the basal plane and an O atom of the nitrato ligand in the apical site. Additional qu­anti­tative information about the structure is given in Fig. 2 ▸, which includes the displacement of each porphyrin core atom (in units of 0.01 Å) from the 24-atom mean plane. Averaged values of the chemically unique bond lengths (in Å) and angles (in °) are also shown. The mean absolute core-atom displacements of *C*_a_, *C*_b_, *C*_m_ and *C*_av_ are 0.11 (2), 0.28 (3), 0.04 (2) and 0.16 (10) Å, respectively, and the monodentate nitrato ligand forms a dihedral angle of 43.69 (13)° with the plane defined by the Mn1, N3 and O1 atoms.

The porphyrin core shows a characteristic saddle-shaped distortion and the Mn1^III^ atom is displaced by 0.22 (4) Å from the 24-atom porphyrin plane in the direction of the nitrato ligand. This value is smaller than the displacement of the iron atom (0.63 Å) in [Fe(TPP)(NO)_3_] reported by Wyllie *et al.* (2007 ▸). This difference is explained by the high-spin configuration of 3*d* ^5^ Fe^III^ where the occupied *d*_(*x*2–*y*2)_ orbital ‘pushes’ the metal out of the porphyrin plane, and the empty *d*_(*x*2–*y*2)_ orbital of 3*d* ^4^ Mn^III^ allows a more in-plane conformation (Suslick & Watson, 1991 ▸).

**Figure 3:**
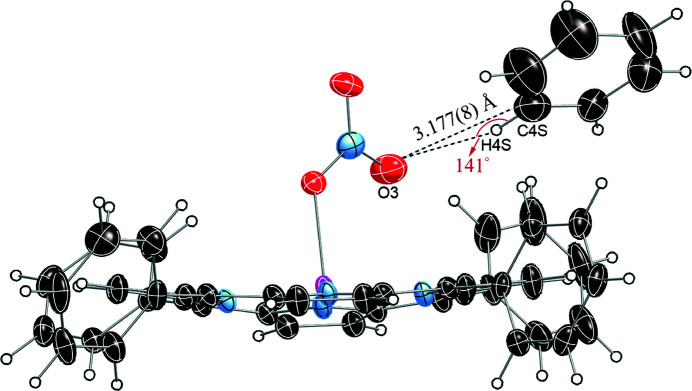
The C—H⋯O inter­actions between the apical nitrato ligand and the benzene solvent mol­ecule.

**Figure 4:**
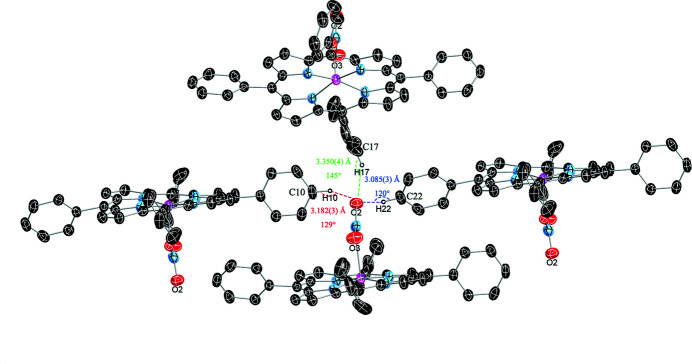
C—H⋯O hydrogen-bonding inter­actions between adjacent porphyrin mol­ecules (dashed lines).

**Figure 5:**
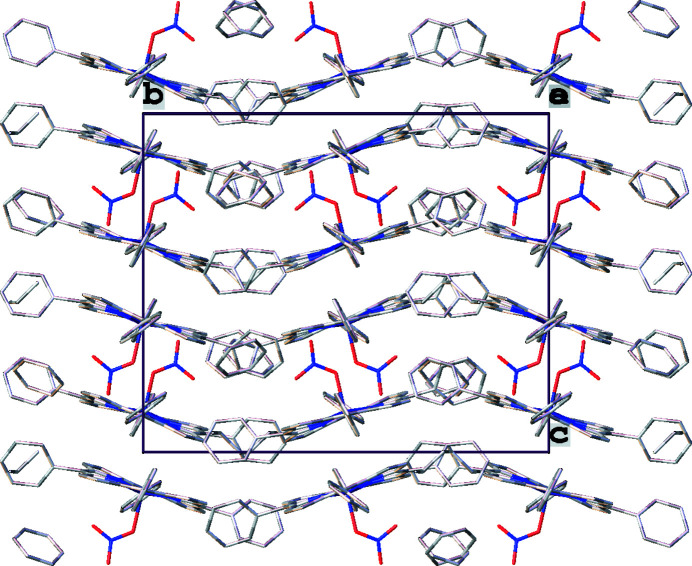
A view of the mol­ecular packing of the title compound in the crystal structure, as seen in a projection along [100]. H atoms have been omitted for clarity.

In the title compound, C—H⋯O hydrogen-bonding inter­actions are found between the disordered benzene solvent mol­ecule (C4*S*) and the apical nitrato ligand (O3), as illus­trated in Fig. 3 ▸ and detailed in Table 2 ▸. Similar hydrogen bonds are also found between the apical ligand and phenyl rings of adjacent porphyrin mol­ecules (Fig. 4 ▸, Table 2 ▸). All these structural parameters are consistent with literature data where C—H⋯O bonds range from 3.00–4.00 Å (Desiraju, 1996 ▸), with angles of 120–180° (Steiner & Desiraju, 1998 ▸). The mol­ecular packing of the title compound is shown in Fig. 5 ▸.

## Synthesis and crystallization

**General information**. All experimental manipulations were performed under a purified nitro­gen atmosphere using Schlenk techniques. Except for the solvent used in column chromatography, all solvents used in the experimental process were treated under dry conditions and exclusion of oxygen. Benzene and *n*-hexane were distilled under argon protection, and then refluxed over sodium/benzo­phenone and potassium–sodium alloy, respectively. All solvents used in the anhydrous and anaerobic operation (Schlenk system) were treated with the pump–freeze–thaw method three times before use.

The title compound was obtained serendipitously in an unsuccessful attempt to isolate the five-coordinate manganese(II) nitrosyl species [Mn(TPP)(NO)]. [Mn(TPP)OH] was prepared according to a reported method (He *et al.*, 2016 ▸). The purple [Mn(TPP)OH] powder (10 mg, 0.0015 mmol) was reduced by ethyl mercaptan for 48 h with benzene as solvent, then the solution was evaporated to dryness. NO gas was then bubbled slowly in a solution of the residue in degassed benzene for 5 minutes under an argon atmosphere. There was a dramatic color change from greenish yellow to red. The red solution was finally layered with hexa­nes. Black, block-shaped crystals were obtained several weeks later.

## Refinement

Crystal data, data collection and structure refinement details are summarized in Table 3 ▸. The benzene mol­ecule is disordered around a twofold rotation axis. Thus, the occupancy of all atoms was constrained to 1/2, and the C4*S*—C9*S* distance constrained to 1.45 Å. One outlier reflection, 332, was omitted from the refinement.

## Supplementary Material

Crystal structure: contains datablock(s) I. DOI: 10.1107/S2414314622003868/wm4162sup1.cif

Structure factors: contains datablock(s) I. DOI: 10.1107/S2414314622003868/wm4162Isup3.hkl

CCDC reference: 2165166

Additional supporting information:  crystallographic information; 3D view; checkCIF report

## Figures and Tables

**Table 1:** Selected structural parameters (Å) for related metalloporphyrin nitrato complexes Δ_4_ is the displacement of the metal atom from the mean plane of the four pyrrole nitro­gen atoms and Δ_24_ is the displacement of the metal atom from the 24-atom mean plane.

Complex	Δ_4_	Δ_24_	*M*—O	N—O1	*M*—N_p_	Ref.
[Mn(TPP)(NO_3_)] (*Pccn*) benzene and *n*-hexane hemisolvate	0.23	0.22	2.1246 (18)	1.260 (3) 1.236 (3) 1.230 (3)	2.011 (6)	This work
[Mn(TPP)(NO_3_)] (*P* 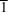 ) benzene disolvate	0.21	0.20	2.101 (3)	1.298 (4) 1.226 (5) 1.226 (5)	2.007 (9)	(Suslick & Watson, 1991 ▸)
[Fe(OEP)(NO_3_)] (*P*2_1_/*c*)	0.40	0.45	1.966 (2)	1.301 (3) 1.199 (3) 1.212 (3)	2.047 (6)	(Wyllie *et al.*, 2007 ▸)
[Fe(OEP)(NO_3_)] (*P* 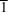 )	0.46	0.50	2.016 (3)	1.206 (5) 1.198 (4) 1.208 (6)	2.056 (1)	(Ellison *et al.*, 1996 ▸)
[Fe(4—Me-TTP)(NO_3_)]	0.47	0.53	1.971 (3)	1.262 (5) 1.252 (5) 1.221 (4)	2.063 (13)	(Bhuyan & Sarkar, 2013 ▸)
[Fe(TPP)(NO_3_)]	0.54	0.63	2.121 (6) 2.19 (10)	1.27 (10) 1.285 (21) 1.217 (3)	2.085 (10)	(Wyllie *et al.*, 2007 ▸)
[Fe(TpivPP)(NO_3_)]	0.42	0.49	2.123 (3) 2.226 (3)	1.271 (4) 1.252 (4) 1.214 (3)	2.070 (16)	(Munro & Scheidt, 1998 ▸)
[Fe(4-OMe-TPP)(NO_3_)]	0.55	0.62	2.169 (5) 2.169 (5)	1.216 (5) 1.276 (8) 1.216 (5)	2.05 (3)	(Bhuyan & Sarkar, 2013 ▸)

**Table 2:** Hydrogen-bond geometry (Å, °)

*D*—H⋯*A*	*D*—H	H⋯*A*	*D*⋯*A*	*D*—H⋯*A*
C4*S*—H4*S*⋯O3	0.95	2.38	3.177 (8)	141
C10—H10⋯O2^i^	0.95	2.50	3.182 (3)	129
C17—H17⋯O2^ii^	0.95	2.53	3.350 (4)	145
C22—H22⋯O2^iii^	0.95	2.49	3.085 (3)	120

Symmetry codes: (i) 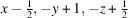 ; (ii) 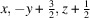 ; (iii) 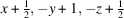 .

**Table 3:** Experimental details

Crystal data	
Chemical formula	[Mn(C_44_H_28_N_4_O_3_)(NO_3_)]·0.5C_6_H_14_·0.5C_6_H_6_
*M*_r_	811.79
Crystal system, space group	Orthorhombic, *P* *c* *c* *n*
Temperature (K)	100
*a*, *b*, *c* (Å)	20.1021 (10), 21.5505 (9), 17.9807 (9)
*V* (Å^3^)	7789.4 (6)
*Z*	8
Radiation type	Mo *K*α
μ (mm^−1^)	0.39
Crystal size (mm)	0.33 × 0.29 × 0.12

Data collection	
Diffractometer	Bruker APEXII CCD
Absorption correction	Multi-scan (*SADABS*; Krause *et al.*, 2015 ▸)
*T*_min_, *T*_max_	0.763, 0.865
No. of measured, independent and observed [*I* > 2σ(*I*)] reflections	58775, 8270, 6163
*R*_int_	0.058
(sin θ/λ)_max_ (Å^−1^)	0.633

Refinement	
*R*[*F* ^2^ > 2σ(*F* ^2^)], *wR*(*F* ^2^), *S*	0.050, 0.148, 1.07
No. of reflections	8270
No. of parameters	560
No. of restraints	37
H-atom treatment	H-atom parameters constrained
Δρ_max_, Δρ_min_ (e Å^−3^)	0.80, −0.48

Computer programs: *APEX2* and *SAINT* (Bruker, 2014 ▸), *SHELXT* (Sheldrick, 2015*a* ▸), *SHELXL* (Sheldrick, 2015*b* ▸), *OLEX2* (Dolomanov *et al.*, 2009 ▸) and *publCIF* (Westrip, 2010 ▸).

## full crystallographic data

### Crystal data

**Table:**

[Mn(C_44_H_28_N_4_O_3_)(NO_3_)]·0.5C_6_H_14_·0.5C_6_H_6_	*D*_x_ = 1.384 Mg m^−^^3^
*M**_r_* = 811.79	Mo *K*α radiation, λ = 0.71073 Å
Orthorhombic, *P**c**c**n*	Cell parameters from 9845 reflections
*a* = 20.1021 (10) Å	θ = 2.5–26.7°
*b* = 21.5505 (9) Å	µ = 0.39 mm^−^^1^
*c* = 17.9807 (9) Å	*T* = 100 K
*V* = 7789.4 (6) Å^3^	Block, black
*Z* = 8	0.33 × 0.29 × 0.12 mm
*F*(000) = 3376	

### Data collection

**Table:**

Bruker APEXII CCD diffractometer	6163 reflections with *I* > 2σ(*I*)
φ and ω scans	*R*_int_ = 0.058
Absorption correction: multi-scan (*SADABS*; Krause *et al.*, 2015)	θ_max_ = 26.7°, θ_min_ = 2.3°
*T*_min_ = 0.763, *T*_max_ = 0.865	*h* = −25→23
58775 measured reflections	*k* = −27→24
8270 independent reflections	*l* = −22→21

### Refinement

**Table:**

Refinement on *F*^2^	Primary atom site location: dual
Least-squares matrix: full	Hydrogen site location: inferred from neighbouring sites
*R*[*F*^2^ > 2σ(*F*^2^)] = 0.050	H-atom parameters constrained
*wR*(*F*^2^) = 0.148	*w* = 1/[σ^2^(*F*_o_^2^) + (0.0686*P*)^2^ + 10.1161*P*] where *P* = (*F*_o_^2^ + 2*F*_c_^2^)/3
*S* = 1.07	(Δ/σ)_max_ = 0.097
8270 reflections	Δρ_max_ = 0.80 e Å^−^^3^
560 parameters	Δρ_min_ = −0.48 e Å^−^^3^
37 restraints	

### Special details

**Table:**

Geometry. All esds (except the esd in the dihedral angle between two l.s. planes) are estimated using the full covariance matrix. The cell esds are taken into account individually in the estimation of esds in distances, angles and torsion angles; correlations between esds in cell parameters are only used when they are defined by crystal symmetry. An approximate (isotropic) treatment of cell esds is used for estimating esds involving l.s. planes.

### Fractional atomic coordinates and isotropic or equivalent isotropic displacement parameters (Å^2^)

**Table:**

	*x*	*y*	*z*	*U*_iso_*/*U*_eq_	Occ. (<1)
Mn1	0.42182 (2)	0.50321 (2)	0.38016 (2)	0.01235 (11)	
O1	0.42711 (8)	0.52498 (8)	0.26507 (10)	0.0206 (4)	
O2	0.41592 (10)	0.58633 (9)	0.17166 (11)	0.0311 (5)	
O3	0.42351 (12)	0.62345 (10)	0.28275 (12)	0.0416 (6)	
N1	0.49166 (9)	0.43691 (8)	0.36975 (11)	0.0144 (4)	
N2	0.35071 (9)	0.43777 (8)	0.36889 (11)	0.0148 (4)	
N3	0.35138 (9)	0.56361 (9)	0.41173 (11)	0.0151 (4)	
N4	0.49211 (9)	0.56102 (8)	0.41874 (11)	0.0147 (4)	
N5	0.42152 (11)	0.57920 (11)	0.23960 (13)	0.0259 (5)	
C1	0.42051 (11)	0.28091 (11)	0.31319 (15)	0.0183 (5)	
C2	0.44918 (13)	0.23571 (11)	0.35761 (16)	0.0259 (6)	
H2	0.467963	0.247013	0.404159	0.031*	
C3	0.45086 (14)	0.17416 (12)	0.33504 (18)	0.0304 (6)	
H3	0.470578	0.143610	0.366108	0.036*	
C4	0.42397 (14)	0.15745 (12)	0.26773 (19)	0.0341 (7)	
H4	0.425615	0.115394	0.251982	0.041*	
C5	0.39436 (16)	0.20192 (13)	0.22261 (18)	0.0369 (7)	
H5	0.375509	0.190271	0.176218	0.044*	
C6	0.39240 (14)	0.26347 (12)	0.24550 (16)	0.0298 (6)	
H6	0.371794	0.293805	0.214858	0.036*	
C7	0.17559 (12)	0.50079 (10)	0.38931 (13)	0.0158 (5)	
C8	0.14138 (12)	0.47327 (11)	0.44853 (15)	0.0198 (5)	
H8	0.165523	0.456573	0.489306	0.024*	
C9	0.07229 (12)	0.47019 (12)	0.44807 (16)	0.0243 (6)	
H9	0.049340	0.451340	0.488358	0.029*	
C10	0.03678 (13)	0.49480 (12)	0.38843 (15)	0.0244 (6)	
H10	−0.010419	0.492704	0.387856	0.029*	
C11	0.07056 (13)	0.52232 (13)	0.33002 (16)	0.0256 (6)	
H11	0.046353	0.539209	0.289402	0.031*	
C12	0.13958 (12)	0.52542 (11)	0.33034 (15)	0.0217 (5)	
H12	0.162274	0.544480	0.290016	0.026*	
C13	0.42453 (11)	0.72160 (11)	0.45726 (15)	0.0189 (5)	
C14	0.45003 (16)	0.75859 (13)	0.4016 (2)	0.0404 (8)	
H14	0.466560	0.740272	0.357213	0.048*	
C15	0.45157 (18)	0.82290 (14)	0.4103 (2)	0.0527 (10)	
H15	0.468977	0.848052	0.371515	0.063*	
C16	0.42877 (14)	0.84987 (13)	0.4729 (2)	0.0398 (8)	
H16	0.429418	0.893761	0.477800	0.048*	
C17	0.40443 (17)	0.81338 (14)	0.5299 (2)	0.0430 (9)	
H17	0.389609	0.832124	0.574726	0.052*	
C18	0.40157 (16)	0.74907 (13)	0.52169 (17)	0.0338 (7)	
H18	0.383801	0.724157	0.560487	0.041*	
C19	0.66708 (12)	0.50305 (10)	0.38165 (13)	0.0154 (5)	
C20	0.70957 (12)	0.45885 (11)	0.41236 (14)	0.0198 (5)	
H20	0.691967	0.426573	0.442483	0.024*	
C21	0.77752 (13)	0.46181 (13)	0.39912 (15)	0.0256 (6)	
H21	0.806087	0.431261	0.419872	0.031*	
C22	0.80410 (13)	0.50902 (13)	0.35582 (16)	0.0271 (6)	
H22	0.850647	0.510865	0.346956	0.032*	
C23	0.76238 (13)	0.55324 (13)	0.32574 (15)	0.0277 (6)	
H23	0.780381	0.585843	0.296427	0.033*	
C24	0.69414 (13)	0.55040 (12)	0.33805 (15)	0.0226 (5)	
H24	0.665777	0.580821	0.316693	0.027*	
C(A1	0.55989 (11)	0.44641 (10)	0.36743 (13)	0.0146 (5)	
C(A2	0.48166 (12)	0.37726 (10)	0.34335 (13)	0.0157 (5)	
C(A3	0.35993 (12)	0.37606 (10)	0.35121 (14)	0.0169 (5)	
C(A4	0.28251 (12)	0.44579 (10)	0.37543 (13)	0.0159 (5)	
C(A5	0.28327 (12)	0.55656 (11)	0.40428 (14)	0.0174 (5)	
C(A6	0.36142 (12)	0.62527 (10)	0.43015 (14)	0.0173 (5)	
C(A7	0.48260 (12)	0.62090 (10)	0.44399 (13)	0.0162 (5)	
C(A8	0.56024 (11)	0.55124 (10)	0.42006 (13)	0.0150 (5)	
C(B1	0.59185 (12)	0.39247 (11)	0.33697 (14)	0.0194 (5)	
H(B1	0.638168	0.387514	0.328321	0.023*	
C(B2	0.54403 (12)	0.35015 (11)	0.32289 (14)	0.0191 (5)	
H(B2	0.550561	0.309748	0.303040	0.023*	
C(B3	0.29657 (12)	0.34533 (11)	0.34796 (15)	0.0216 (5)	
H(B3	0.289310	0.302652	0.337501	0.026*	
C(B4	0.24941 (12)	0.38788 (11)	0.36239 (15)	0.0213 (5)	
H(B4	0.202762	0.380878	0.363712	0.026*	
C(B5	0.25083 (13)	0.61474 (11)	0.41847 (15)	0.0230 (6)	
H(B5	0.204257	0.622200	0.417728	0.028*	
C(B6	0.29864 (12)	0.65671 (11)	0.43303 (16)	0.0230 (6)	
H(B6	0.291982	0.699496	0.443361	0.028*	
C(B7	0.54509 (12)	0.64704 (11)	0.46568 (14)	0.0182 (5)	
H(B7	0.551968	0.687212	0.486043	0.022*	
C(B8	0.59258 (12)	0.60410 (11)	0.45188 (14)	0.0176 (5)	
H(B8	0.638833	0.608255	0.461525	0.021*	
C(M1	0.42063 (12)	0.34755 (10)	0.33679 (13)	0.0162 (5)	
C(M2	0.25010 (12)	0.50150 (10)	0.38919 (13)	0.0153 (5)	
C(M3	0.42232 (11)	0.65274 (10)	0.44520 (14)	0.0168 (5)	
C(M4	0.59319 (12)	0.49958 (10)	0.39124 (13)	0.0145 (5)	
C1S	0.28678 (18)	0.23978 (17)	0.4985 (2)	0.0488 (9)	
H1SA	0.315264	0.276355	0.508279	0.059*	
H1SB	0.298393	0.223535	0.448647	0.059*	
C2S	0.30152 (18)	0.18932 (17)	0.5575 (2)	0.0509 (9)	
H2SA	0.289087	0.204976	0.607336	0.061*	
H2SB	0.274282	0.152072	0.546955	0.061*	
C3S	0.37423 (19)	0.17186 (17)	0.5572 (2)	0.0571 (10)	
H3SA	0.387512	0.159787	0.506761	0.086*	
H3SB	0.381507	0.136996	0.591202	0.086*	
H3SC	0.400895	0.207461	0.573341	0.086*	
C4S	0.3238 (4)	0.7350 (3)	0.2525 (4)	0.0454 (15)	0.5
H4S	0.363543	0.718301	0.272952	0.054*	0.5
C5S	0.2861 (11)	0.7015 (10)	0.2020 (13)	0.069 (6)	0.5
H5S	0.299310	0.660302	0.190509	0.083*	0.5
C6S	0.2325 (5)	0.7245 (4)	0.1690 (6)	0.077 (3)	0.5
H6S	0.210217	0.701910	0.131113	0.093*	0.5
C7S	0.2079 (11)	0.7868 (11)	0.1930 (17)	0.075 (6)	0.5
H7S	0.167838	0.803904	0.173935	0.090*	0.5
C8S	0.2462 (4)	0.8181 (4)	0.2438 (5)	0.0518 (19)	0.5
H8S	0.233013	0.858391	0.259270	0.062*	0.5
C9S	0.3018 (3)	0.7930 (3)	0.2721 (4)	0.0414 (15)	0.5
H9S	0.326862	0.816292	0.307085	0.050*	0.5

### Atomic displacement parameters (Å^2^)

**Table:**

	*U*^11^	*U*^22^	*U*^33^	*U*^12^	*U*^13^	*U*^23^
Mn1	0.01075 (19)	0.00928 (18)	0.0170 (2)	0.00011 (12)	−0.00051 (13)	−0.00002 (13)
O1	0.0168 (9)	0.0267 (9)	0.0183 (9)	0.0015 (7)	−0.0016 (7)	0.0008 (7)
O2	0.0329 (11)	0.0353 (11)	0.0252 (11)	−0.0007 (8)	−0.0020 (8)	0.0095 (9)
O3	0.0610 (15)	0.0309 (11)	0.0328 (12)	−0.0039 (10)	0.0025 (10)	−0.0002 (9)
N1	0.0149 (10)	0.0101 (9)	0.0181 (10)	−0.0004 (7)	−0.0014 (8)	−0.0002 (8)
N2	0.0134 (10)	0.0104 (9)	0.0206 (11)	−0.0001 (7)	0.0007 (8)	0.0002 (8)
N3	0.0117 (9)	0.0115 (9)	0.0220 (11)	−0.0004 (7)	0.0000 (8)	0.0009 (8)
N4	0.0149 (10)	0.0100 (9)	0.0191 (11)	0.0013 (7)	0.0001 (8)	0.0005 (8)
N5	0.0201 (11)	0.0309 (12)	0.0268 (13)	−0.0047 (9)	0.0010 (9)	−0.0002 (10)
C1	0.0126 (11)	0.0137 (11)	0.0286 (14)	−0.0017 (9)	0.0024 (10)	−0.0036 (10)
C2	0.0255 (14)	0.0180 (12)	0.0342 (15)	0.0028 (10)	−0.0041 (12)	−0.0024 (11)
C3	0.0262 (14)	0.0138 (12)	0.0511 (19)	0.0033 (10)	−0.0036 (13)	−0.0017 (12)
C4	0.0252 (14)	0.0164 (13)	0.061 (2)	0.0002 (11)	−0.0031 (14)	−0.0159 (13)
C5	0.0428 (18)	0.0290 (15)	0.0390 (18)	−0.0024 (13)	−0.0086 (14)	−0.0149 (13)
C6	0.0345 (15)	0.0233 (14)	0.0317 (16)	0.0017 (11)	−0.0060 (13)	−0.0038 (11)
C7	0.0138 (11)	0.0128 (11)	0.0209 (12)	−0.0001 (8)	0.0010 (9)	−0.0044 (9)
C8	0.0176 (12)	0.0173 (12)	0.0247 (13)	−0.0006 (9)	−0.0012 (10)	0.0000 (10)
C9	0.0196 (13)	0.0219 (13)	0.0313 (15)	−0.0043 (10)	0.0077 (11)	−0.0043 (11)
C10	0.0120 (12)	0.0269 (14)	0.0343 (16)	−0.0011 (10)	0.0009 (10)	−0.0107 (11)
C11	0.0172 (13)	0.0303 (14)	0.0293 (15)	0.0033 (10)	−0.0036 (11)	−0.0083 (12)
C12	0.0183 (13)	0.0225 (12)	0.0243 (14)	0.0032 (10)	0.0001 (10)	0.0005 (10)
C13	0.0128 (11)	0.0130 (11)	0.0309 (14)	0.0013 (9)	−0.0068 (10)	−0.0024 (10)
C14	0.0412 (18)	0.0175 (14)	0.062 (2)	0.0008 (12)	0.0178 (16)	0.0018 (14)
C15	0.044 (2)	0.0170 (14)	0.097 (3)	−0.0012 (13)	0.024 (2)	0.0097 (17)
C16	0.0225 (14)	0.0127 (12)	0.084 (3)	0.0005 (11)	−0.0136 (15)	−0.0049 (15)
C17	0.052 (2)	0.0296 (16)	0.048 (2)	0.0194 (14)	−0.0259 (16)	−0.0189 (15)
C18	0.0496 (18)	0.0196 (13)	0.0321 (16)	0.0122 (12)	−0.0080 (14)	−0.0017 (12)
C19	0.0130 (11)	0.0159 (11)	0.0173 (12)	−0.0024 (9)	0.0003 (9)	−0.0027 (9)
C20	0.0194 (13)	0.0184 (12)	0.0216 (13)	0.0013 (9)	−0.0010 (10)	−0.0002 (10)
C21	0.0183 (13)	0.0278 (14)	0.0308 (15)	0.0066 (10)	−0.0019 (11)	−0.0055 (11)
C22	0.0155 (13)	0.0340 (15)	0.0317 (15)	−0.0039 (10)	0.0045 (11)	−0.0089 (12)
C23	0.0241 (14)	0.0313 (14)	0.0276 (14)	−0.0080 (11)	0.0063 (11)	0.0013 (12)
C24	0.0212 (13)	0.0217 (13)	0.0249 (14)	−0.0017 (10)	−0.0010 (10)	0.0026 (10)
C(A1	0.0141 (11)	0.0130 (11)	0.0167 (12)	0.0011 (8)	0.0011 (9)	0.0022 (9)
C(A2	0.0184 (12)	0.0117 (10)	0.0171 (12)	0.0026 (9)	−0.0004 (9)	0.0006 (9)
C(A3	0.0170 (12)	0.0122 (11)	0.0214 (13)	−0.0010 (9)	−0.0021 (10)	0.0002 (9)
C(A4	0.0148 (11)	0.0143 (11)	0.0185 (12)	−0.0012 (9)	−0.0007 (9)	0.0010 (9)
C(A5	0.0150 (12)	0.0139 (11)	0.0232 (13)	0.0020 (9)	0.0011 (10)	0.0011 (9)
C(A6	0.0165 (12)	0.0126 (11)	0.0227 (13)	0.0011 (9)	0.0008 (10)	0.0011 (9)
C(A7	0.0167 (12)	0.0127 (11)	0.0191 (12)	0.0001 (9)	−0.0012 (9)	−0.0002 (9)
C(A8	0.0132 (11)	0.0139 (11)	0.0180 (12)	−0.0005 (8)	−0.0014 (9)	0.0016 (9)
C(B1	0.0176 (12)	0.0161 (11)	0.0245 (14)	0.0023 (9)	0.0023 (10)	−0.0003 (10)
C(B2	0.0182 (12)	0.0133 (11)	0.0258 (13)	0.0031 (9)	0.0009 (10)	−0.0029 (10)
C(B3	0.0175 (12)	0.0138 (11)	0.0336 (15)	−0.0041 (9)	−0.0002 (11)	−0.0024 (10)
C(B4	0.0149 (12)	0.0170 (11)	0.0320 (15)	−0.0030 (9)	0.0005 (10)	−0.0003 (10)
C(B5	0.0147 (12)	0.0158 (11)	0.0386 (16)	0.0022 (9)	−0.0011 (11)	−0.0022 (11)
C(B6	0.0162 (12)	0.0141 (11)	0.0386 (16)	0.0024 (9)	−0.0017 (11)	−0.0025 (11)
C(B7	0.0192 (12)	0.0131 (11)	0.0224 (13)	−0.0021 (9)	−0.0011 (10)	−0.0018 (9)
C(B8	0.0156 (12)	0.0170 (11)	0.0202 (13)	−0.0011 (9)	−0.0021 (9)	−0.0007 (9)
C(M1	0.0179 (12)	0.0112 (10)	0.0196 (13)	0.0007 (9)	−0.0016 (9)	0.0001 (9)
C(M2	0.0135 (11)	0.0149 (11)	0.0175 (12)	0.0002 (9)	−0.0002 (9)	0.0025 (9)
C(M3	0.0173 (12)	0.0114 (10)	0.0215 (13)	0.0006 (9)	−0.0012 (10)	−0.0003 (9)
C(M4	0.0119 (11)	0.0145 (11)	0.0170 (12)	0.0007 (8)	−0.0007 (8)	0.0019 (9)
C1S	0.059 (2)	0.0438 (19)	0.043 (2)	0.0006 (17)	−0.0063 (17)	−0.0030 (16)
C2S	0.049 (2)	0.046 (2)	0.057 (2)	−0.0065 (16)	0.0016 (18)	−0.0077 (17)
C3S	0.060 (2)	0.045 (2)	0.067 (3)	−0.0008 (18)	−0.001 (2)	−0.0046 (18)
C4S	0.050 (4)	0.048 (4)	0.038 (4)	0.001 (3)	0.003 (3)	0.004 (3)
C5S	0.074 (10)	0.046 (7)	0.087 (12)	0.001 (6)	−0.014 (9)	−0.015 (8)
C6S	0.068 (6)	0.060 (5)	0.104 (8)	−0.016 (4)	−0.024 (5)	−0.012 (5)
C7S	0.046 (7)	0.077 (9)	0.103 (11)	0.010 (6)	−0.043 (7)	−0.015 (8)
C8S	0.044 (4)	0.053 (4)	0.059 (5)	−0.001 (3)	−0.006 (4)	−0.011 (4)
C9S	0.042 (3)	0.044 (3)	0.038 (4)	0.004 (3)	0.006 (3)	−0.001 (3)

### Geometric parameters (Å, º)

**Table:**

Mn1—O1	2.1246 (18)	C21—H21	0.9500
Mn1—N1	2.0119 (19)	C21—C22	1.388 (4)
Mn1—N2	2.0184 (19)	C22—H22	0.9500
Mn1—N3	2.0052 (19)	C22—C23	1.380 (4)
Mn1—N4	2.0074 (19)	C23—H23	0.9500
O1—N5	1.260 (3)	C23—C24	1.391 (4)
O2—N5	1.236 (3)	C24—H24	0.9500
O3—N5	1.230 (3)	C(A1—C(B1	1.437 (3)
N1—C(A1	1.387 (3)	C(A1—C(M4	1.394 (3)
N1—C(A2	1.385 (3)	C(A2—C(B2	1.431 (3)
N2—C(A3	1.380 (3)	C(A2—C(M1	1.389 (3)
N2—C(A4	1.387 (3)	C(A3—C(B3	1.437 (3)
N3—C(A5	1.384 (3)	C(A3—C(M1	1.391 (3)
N3—C(A6	1.384 (3)	C(A4—C(B4	1.434 (3)
N4—C(A7	1.381 (3)	C(A4—C(M2	1.388 (3)
N4—C(A8	1.386 (3)	C(A5—C(B5	1.436 (3)
C1—C2	1.385 (4)	C(A5—C(M2	1.388 (3)
C1—C6	1.394 (4)	C(A6—C(B6	1.433 (3)
C1—C(M1	1.497 (3)	C(A6—C(M3	1.386 (3)
C2—H2	0.9500	C(A7—C(B7	1.431 (3)
C2—C3	1.388 (3)	C(A7—C(M3	1.393 (3)
C3—H3	0.9500	C(A8—C(B8	1.431 (3)
C3—C4	1.374 (4)	C(A8—C(M4	1.395 (3)
C4—H4	0.9500	C(B1—H(B1	0.9500
C4—C5	1.390 (4)	C(B1—C(B2	1.349 (3)
C5—H5	0.9500	C(B2—H(B2	0.9500
C5—C6	1.389 (4)	C(B3—H(B3	0.9500
C6—H6	0.9500	C(B3—C(B4	1.344 (3)
C7—C8	1.399 (3)	C(B4—H(B4	0.9500
C7—C12	1.389 (3)	C(B5—H(B5	0.9500
C7—C(M2	1.498 (3)	C(B5—C(B6	1.346 (3)
C8—H8	0.9500	C(B6—H(B6	0.9500
C8—C9	1.391 (3)	C(B7—H(B7	0.9500
C9—H9	0.9500	C(B7—C(B8	1.353 (3)
C9—C10	1.393 (4)	C(B8—H(B8	0.9500
C10—H10	0.9500	C1S—C1S^i^	1.543 (7)
C10—C11	1.384 (4)	C1S—H1SA	0.9900
C11—H11	0.9500	C1S—H1SB	0.9900
C11—C12	1.389 (4)	C1S—C2S	1.548 (5)
C12—H12	0.9500	C2S—H2SA	0.9900
C13—C14	1.379 (4)	C2S—H2SB	0.9900
C13—C18	1.380 (4)	C2S—C3S	1.509 (5)
C13—C(M3	1.500 (3)	C3S—H3SA	0.9800
C14—H14	0.9500	C3S—H3SB	0.9800
C14—C15	1.395 (4)	C3S—H3SC	0.9800
C15—H15	0.9500	C4S—H4S	0.9500
C15—C16	1.347 (5)	C4S—C5S	1.38 (2)
C16—H16	0.9500	C4S—C9S	1.372 (9)
C16—C17	1.381 (5)	C5S—H5S	0.9500
C17—H17	0.9500	C5S—C6S	1.33 (2)
C17—C18	1.395 (4)	C6S—H6S	0.9500
C18—H18	0.9500	C6S—C7S	1.49 (3)
C19—C20	1.393 (3)	C7S—H7S	0.9500
C19—C24	1.397 (3)	C7S—C8S	1.37 (2)
C19—C(M4	1.497 (3)	C8S—H8S	0.9500
C20—H20	0.9500	C8S—C9S	1.342 (10)
C20—C21	1.388 (4)	C9S—H9S	0.9500
			
N1—Mn1—O1	91.79 (7)	C23—C24—H24	119.8
N1—Mn1—N2	89.34 (8)	N1—C(A1—C(B1	109.5 (2)
N2—Mn1—O1	95.27 (7)	N1—C(A1—C(M4	125.9 (2)
N3—Mn1—O1	99.66 (7)	C(M4—C(A1—C(B1	124.6 (2)
N3—Mn1—N1	168.53 (8)	N1—C(A2—C(B2	109.9 (2)
N3—Mn1—N2	88.96 (8)	N1—C(A2—C(M1	125.8 (2)
N3—Mn1—N4	89.79 (8)	C(M1—C(A2—C(B2	124.3 (2)
N4—Mn1—O1	99.46 (7)	N2—C(A3—C(B3	109.5 (2)
N4—Mn1—N1	88.95 (8)	N2—C(A3—C(M1	125.9 (2)
N4—Mn1—N2	165.21 (8)	C(M1—C(A3—C(B3	124.5 (2)
N5—O1—Mn1	123.66 (15)	N2—C(A4—C(B4	109.7 (2)
C(A1—N1—Mn1	126.01 (15)	N2—C(A4—C(M2	125.9 (2)
C(A2—N1—Mn1	126.15 (15)	C(M2—C(A4—C(B4	124.3 (2)
C(A2—N1—C(A1	105.67 (18)	N3—C(A5—C(B5	109.7 (2)
C(A3—N2—Mn1	126.98 (16)	N3—C(A5—C(M2	126.0 (2)
C(A3—N2—C(A4	105.81 (19)	C(M2—C(A5—C(B5	124.3 (2)
C(A4—N2—Mn1	127.19 (15)	N3—C(A6—C(B6	109.5 (2)
C(A5—N3—Mn1	126.86 (16)	N3—C(A6—C(M3	125.8 (2)
C(A6—N3—Mn1	126.00 (15)	C(M3—C(A6—C(B6	124.6 (2)
C(A6—N3—C(A5	105.82 (18)	N4—C(A7—C(B7	109.6 (2)
C(A7—N4—Mn1	126.58 (15)	N4—C(A7—C(M3	125.9 (2)
C(A7—N4—C(A8	105.85 (18)	C(M3—C(A7—C(B7	124.4 (2)
C(A8—N4—Mn1	127.38 (15)	N4—C(A8—C(B8	109.6 (2)
O2—N5—O1	118.8 (2)	N4—C(A8—C(M4	125.7 (2)
O3—N5—O1	119.1 (2)	C(M4—C(A8—C(B8	124.6 (2)
O3—N5—O2	122.0 (2)	C(A1—C(B1—H(B1	126.3
C2—C1—C6	118.9 (2)	C(B2—C(B1—C(A1	107.5 (2)
C2—C1—C(M1	120.7 (2)	C(B2—C(B1—H(B1	126.3
C6—C1—C(M1	120.5 (2)	C(A2—C(B2—H(B2	126.3
C1—C2—H2	119.5	C(B1—C(B2—C(A2	107.4 (2)
C1—C2—C3	120.9 (3)	C(B1—C(B2—H(B2	126.3
C3—C2—H2	119.5	C(A3—C(B3—H(B3	126.2
C2—C3—H3	120.0	C(B4—C(B3—C(A3	107.6 (2)
C4—C3—C2	119.9 (3)	C(B4—C(B3—H(B3	126.2
C4—C3—H3	120.0	C(A4—C(B4—H(B4	126.3
C3—C4—H4	119.9	C(B3—C(B4—C(A4	107.3 (2)
C3—C4—C5	120.1 (2)	C(B3—C(B4—H(B4	126.3
C5—C4—H4	119.9	C(A5—C(B5—H(B5	126.4
C4—C5—H5	120.1	C(B6—C(B5—C(A5	107.3 (2)
C6—C5—C4	119.9 (3)	C(B6—C(B5—H(B5	126.4
C6—C5—H5	120.1	C(A6—C(B6—H(B6	126.1
C1—C6—H6	119.8	C(B5—C(B6—C(A6	107.7 (2)
C5—C6—C1	120.3 (3)	C(B5—C(B6—H(B6	126.2
C5—C6—H6	119.8	C(A7—C(B7—H(B7	126.3
C8—C7—C(M2	119.8 (2)	C(B8—C(B7—C(A7	107.5 (2)
C12—C7—C8	119.1 (2)	C(B8—C(B7—H(B7	126.3
C12—C7—C(M2	121.1 (2)	C(A8—C(B8—H(B8	126.3
C7—C8—H8	119.8	C(B7—C(B8—C(A8	107.3 (2)
C9—C8—C7	120.4 (2)	C(B7—C(B8—H(B8	126.3
C9—C8—H8	119.8	C(A2—C(M1—C1	117.9 (2)
C8—C9—H9	120.1	C(A2—C(M1—C(A3	123.7 (2)
C8—C9—C10	119.9 (2)	C(A3—C(M1—C1	118.4 (2)
C10—C9—H9	120.1	C(A4—C(M2—C7	117.4 (2)
C9—C10—H10	120.1	C(A5—C(M2—C7	119.3 (2)
C11—C10—C9	119.7 (2)	C(A5—C(M2—C(A4	123.3 (2)
C11—C10—H10	120.1	C(A6—C(M3—C13	118.5 (2)
C10—C11—H11	119.8	C(A6—C(M3—C(A7	123.7 (2)
C10—C11—C12	120.5 (3)	C(A7—C(M3—C13	117.6 (2)
C12—C11—H11	119.8	C(A1—C(M4—C19	118.8 (2)
C7—C12—H12	119.8	C(A1—C(M4—C(A8	122.8 (2)
C11—C12—C7	120.3 (2)	C(A8—C(M4—C19	118.3 (2)
C11—C12—H12	119.8	C1S^i^—C1S—H1SA	109.1
C14—C13—C18	119.1 (2)	C1S^i^—C1S—H1SB	109.1
C14—C13—C(M3	118.5 (2)	C1S^i^—C1S—C2S	112.6 (3)
C18—C13—C(M3	122.4 (2)	H1SA—C1S—H1SB	107.8
C13—C14—H14	120.0	C2S—C1S—H1SA	109.1
C13—C14—C15	120.1 (3)	C2S—C1S—H1SB	109.1
C15—C14—H14	120.0	C1S—C2S—H2SA	109.4
C14—C15—H15	119.5	C1S—C2S—H2SB	109.4
C16—C15—C14	121.0 (3)	H2SA—C2S—H2SB	108.0
C16—C15—H15	119.5	C3S—C2S—C1S	111.0 (3)
C15—C16—H16	120.2	C3S—C2S—H2SA	109.4
C15—C16—C17	119.6 (3)	C3S—C2S—H2SB	109.4
C17—C16—H16	120.2	C2S—C3S—H3SA	109.5
C16—C17—H17	119.9	C2S—C3S—H3SB	109.5
C16—C17—C18	120.1 (3)	C2S—C3S—H3SC	109.5
C18—C17—H17	119.9	H3SA—C3S—H3SB	109.5
C13—C18—C17	120.1 (3)	H3SA—C3S—H3SC	109.5
C13—C18—H18	120.0	H3SB—C3S—H3SC	109.5
C17—C18—H18	120.0	C5S—C4S—H4S	121.1
C20—C19—C24	118.9 (2)	C5S—C4S—C9S	117.8 (11)
C20—C19—C(M4	121.9 (2)	C9S—C4S—H4S	121.1
C24—C19—C(M4	119.2 (2)	C4S—C5S—H5S	118.5
C19—C20—H20	119.9	C6S—C5S—C4S	122.9 (16)
C21—C20—C19	120.3 (2)	C6S—C5S—H5S	118.6
C21—C20—H20	119.9	C5S—C6S—H6S	120.8
C20—C21—H21	119.7	C5S—C6S—C7S	118.4 (12)
C22—C21—C20	120.6 (2)	C7S—C6S—H6S	120.8
C22—C21—H21	119.7	C6S—C7S—H7S	121.6
C21—C22—H22	120.3	C8S—C7S—C6S	116.7 (14)
C23—C22—C21	119.5 (2)	C8S—C7S—H7S	121.7
C23—C22—H22	120.3	C7S—C8S—H8S	119.3
C22—C23—H23	119.8	C9S—C8S—C7S	121.5 (11)
C22—C23—C24	120.4 (2)	C9S—C8S—H8S	119.3
C24—C23—H23	119.8	C4S—C9S—H9S	118.7
C19—C24—H24	119.8	C8S—C9S—C4S	122.6 (7)
C23—C24—C19	120.4 (2)	C8S—C9S—H9S	118.7
			
Mn1—O1—N5—O2	−169.95 (16)	C18—C13—C(M3—C(A7	−110.9 (3)
Mn1—O1—N5—O3	11.9 (3)	C19—C20—C21—C22	0.6 (4)
Mn1—N1—C(A1—C(B1	−162.14 (16)	C20—C19—C24—C23	−0.1 (4)
Mn1—N1—C(A1—C(M4	18.3 (3)	C20—C19—C(M4—C(A1	−57.4 (3)
Mn1—N1—C(A2—C(B2	162.58 (16)	C20—C19—C(M4—C(A8	125.9 (2)
Mn1—N1—C(A2—C(M1	−17.0 (3)	C20—C21—C22—C23	−0.1 (4)
Mn1—N2—C(A3—C(B3	−179.46 (17)	C21—C22—C23—C24	−0.6 (4)
Mn1—N2—C(A3—C(M1	−0.9 (4)	C22—C23—C24—C19	0.6 (4)
Mn1—N2—C(A4—C(B4	179.21 (16)	C24—C19—C20—C21	−0.5 (4)
Mn1—N2—C(A4—C(M2	1.9 (4)	C24—C19—C(M4—C(A1	119.8 (2)
Mn1—N3—C(A5—C(B5	−167.60 (17)	C24—C19—C(M4—C(A8	−56.8 (3)
Mn1—N3—C(A5—C(M2	15.5 (4)	C(A1—N1—C(A2—C(B2	−1.3 (3)
Mn1—N3—C(A6—C(B6	166.81 (17)	C(A1—N1—C(A2—C(M1	179.1 (2)
Mn1—N3—C(A6—C(M3	−14.5 (4)	C(A1—C(B1—C(B2—C(A2	0.8 (3)
Mn1—N4—C(A7—C(B7	−179.11 (16)	C(A2—N1—C(A1—C(B1	1.8 (3)
Mn1—N4—C(A7—C(M3	−2.0 (4)	C(A2—N1—C(A1—C(M4	−177.7 (2)
Mn1—N4—C(A8—C(B8	179.77 (16)	C(A3—N2—C(A4—C(B4	0.7 (3)
Mn1—N4—C(A8—C(M4	3.1 (3)	C(A3—N2—C(A4—C(M2	−176.7 (2)
N1—C(A1—C(B1—C(B2	−1.6 (3)	C(A3—C(B3—C(B4—C(A4	−0.4 (3)
N1—C(A1—C(M4—C19	−177.4 (2)	C(A4—N2—C(A3—C(B3	−0.9 (3)
N1—C(A1—C(M4—C(A8	−1.0 (4)	C(A4—N2—C(A3—C(M1	177.6 (2)
N1—C(A2—C(B2—C(B1	0.4 (3)	C(A5—N3—C(A6—C(B6	−0.8 (3)
N1—C(A2—C(M1—C1	−174.8 (2)	C(A5—N3—C(A6—C(M3	177.9 (2)
N1—C(A2—C(M1—C(A3	4.9 (4)	C(A5—C(B5—C(B6—C(A6	−1.5 (3)
N2—C(A3—C(B3—C(B4	0.9 (3)	C(A6—N3—C(A5—C(B5	−0.1 (3)
N2—C(A3—C(M1—C1	−175.7 (2)	C(A6—N3—C(A5—C(M2	−177.1 (2)
N2—C(A3—C(M1—C(A2	4.6 (4)	C(A7—N4—C(A8—C(B8	4.5 (3)
N2—C(A4—C(B4—C(B3	−0.2 (3)	C(A7—N4—C(A8—C(M4	−172.3 (2)
N2—C(A4—C(M2—C7	176.1 (2)	C(A7—C(B7—C(B8—C(A8	1.1 (3)
N2—C(A4—C(M2—C(A5	−5.4 (4)	C(A8—N4—C(A7—C(B7	−3.7 (3)
N3—C(A5—C(B5—C(B6	1.1 (3)	C(A8—N4—C(A7—C(M3	173.4 (2)
N3—C(A5—C(M2—C7	174.8 (2)	C(B1—C(A1—C(M4—C19	3.1 (3)
N3—C(A5—C(M2—C(A4	−3.7 (4)	C(B1—C(A1—C(M4—C(A8	179.5 (2)
N3—C(A6—C(B6—C(B5	1.5 (3)	C(B2—C(A2—C(M1—C1	5.7 (4)
N3—C(A6—C(M3—C13	174.4 (2)	C(B2—C(A2—C(M1—C(A3	−174.6 (2)
N3—C(A6—C(M3—C(A7	−0.3 (4)	C(B3—C(A3—C(M1—C1	2.6 (4)
N4—C(A7—C(B7—C(B8	1.6 (3)	C(B3—C(A3—C(M1—C(A2	−177.1 (2)
N4—C(A7—C(M3—C13	−165.7 (2)	C(B4—C(A4—C(M2—C7	−0.9 (4)
N4—C(A7—C(M3—C(A6	9.0 (4)	C(B4—C(A4—C(M2—C(A5	177.6 (2)
N4—C(A8—C(B8—C(B7	−3.5 (3)	C(B5—C(A5—C(M2—C7	−1.7 (4)
N4—C(A8—C(M4—C19	166.2 (2)	C(B5—C(A5—C(M2—C(A4	179.8 (2)
N4—C(A8—C(M4—C(A1	−10.3 (4)	C(B6—C(A6—C(M3—C13	−7.1 (4)
C1—C2—C3—C4	0.2 (4)	C(B6—C(A6—C(M3—C(A7	178.2 (2)
C2—C1—C6—C5	−1.2 (4)	C(B7—C(A7—C(M3—C13	11.0 (4)
C2—C1—C(M1—C(A2	64.2 (3)	C(B7—C(A7—C(M3—C(A6	−174.2 (2)
C2—C1—C(M1—C(A3	−115.5 (3)	C(B8—C(A8—C(M4—C19	−10.0 (4)
C2—C3—C4—C5	−0.8 (5)	C(B8—C(A8—C(M4—C(A1	173.5 (2)
C3—C4—C5—C6	0.4 (5)	C(M1—C1—C2—C3	−178.2 (2)
C4—C5—C6—C1	0.6 (5)	C(M1—C1—C6—C5	177.8 (3)
C6—C1—C2—C3	0.8 (4)	C(M1—C(A2—C(B2—C(B1	180.0 (2)
C6—C1—C(M1—C(A2	−114.8 (3)	C(M1—C(A3—C(B3—C(B4	−177.7 (2)
C6—C1—C(M1—C(A3	65.5 (3)	C(M2—C7—C8—C9	−177.6 (2)
C7—C8—C9—C10	−0.2 (4)	C(M2—C7—C12—C11	177.6 (2)
C8—C7—C12—C11	−0.5 (4)	C(M2—C(A4—C(B4—C(B3	177.2 (2)
C8—C7—C(M2—C(A4	72.7 (3)	C(M2—C(A5—C(B5—C(B6	178.1 (2)
C8—C7—C(M2—C(A5	−105.9 (3)	C(M3—C13—C14—C15	178.7 (3)
C8—C9—C10—C11	−0.1 (4)	C(M3—C13—C18—C17	−179.6 (3)
C9—C10—C11—C12	0.2 (4)	C(M3—C(A6—C(B6—C(B5	−177.3 (3)
C10—C11—C12—C7	0.1 (4)	C(M3—C(A7—C(B7—C(B8	−175.6 (2)
C12—C7—C8—C9	0.5 (3)	C(M4—C19—C20—C21	176.7 (2)
C12—C7—C(M2—C(A4	−105.3 (3)	C(M4—C19—C24—C23	−177.4 (2)
C12—C7—C(M2—C(A5	76.1 (3)	C(M4—C(A1—C(B1—C(B2	177.9 (2)
C13—C14—C15—C16	0.4 (6)	C(M4—C(A8—C(B8—C(B7	173.2 (2)
C14—C13—C18—C17	−0.1 (4)	C1S^i^—C1S—C2S—C3S	−178.4 (3)
C14—C13—C(M3—C(A6	−105.4 (3)	C4S—C5S—C6S—C7S	−6 (3)
C14—C13—C(M3—C(A7	69.6 (3)	C5S—C4S—C9S—C8S	−0.6 (15)
C14—C15—C16—C17	1.0 (5)	C5S—C6S—C7S—C8S	5 (3)
C15—C16—C17—C18	−2.0 (5)	C6S—C7S—C8S—C9S	−2 (3)
C16—C17—C18—C13	1.5 (5)	C7S—C8S—C9S—C4S	−0.2 (19)
C18—C13—C14—C15	−0.8 (5)	C9S—C4S—C5S—C6S	4 (3)
C18—C13—C(M3—C(A6	74.1 (3)		

Symmetry code: (i) −*x*+1/2, −*y*+1/2, *z*.

### Hydrogen-bond geometry (Å, º)

**Table:**

*D*—H···*A*	*D*—H	H···*A*	*D*···*A*	*D*—H···*A*
C4*S*—H4*S*···O3	0.95	2.38	3.177 (8)	141
C10—H10···O2^ii^	0.95	2.50	3.182 (3)	129
C17—H17···O2^iii^	0.95	2.53	3.350 (4)	145
C22—H22···O2^iv^	0.95	2.49	3.085 (3)	120

Symmetry codes: (ii) *x*−1/2, −*y*+1, −*z*+1/2; (iii) *x*, −*y*+3/2, *z*+1/2; (iv) *x*+1/2, −*y*+1, −*z*+1/2.
